# Time esophageal pH < 4 overestimates the prevalence of pathologic esophageal reflux in subjects with gastroesophageal reflux disease treated with proton pump inhibitors

**DOI:** 10.1186/1471-230X-8-15

**Published:** 2008-05-23

**Authors:** Lauren B Gerson, George Triadafilopoulos, Peyman Sahbaie, Winston Young, Sheldon Sloan, Malcolm Robinson, Philip B Miner, Jerry D Gardner

**Affiliations:** 1 Stanford University School of Medicine, Stanford, CA, USA; 2 Veterans Affairs Palo Alto Health Care System, Palo Alto, CA, USA; 3 Blossomtech, Inc., Apex, NC, USA; 4 Janssen Pharmaceutica Inc., Titusville, NJ, USA; 5 Oklahoma Foundation for Digestive Research, University of Oklahoma Health Sciences Center, Oklahoma City, OK, USA; 6 Science for Organizations, Inc., Mill Valley, CA, USA

## Abstract

**Background:**

A Stanford University study reported that in asymptomatic GERD patients who were being treated with a proton pump inhibitor (PPI), 50% had pathologic esophageal acid exposure.

**Aim:**

We considered the possibility that the high prevalence of pathologic esophageal reflux might simply have resulted from calculating acidity as time pH < 4.

**Methods:**

We calculated integrated acidity and time pH < 4 from the 49 recordings of 24-hour gastric and esophageal pH from the Stanford study as well as from another study of 57 GERD subjects, 26 of whom were treated for 8 days with 20 mg omeprazole or 20 mg rabeprazole in a 2-way crossover fashion.

**Results:**

The prevalence of pathologic 24-hour esophageal reflux in both studies was significantly higher when measured as time pH < 4 than when measured as integrated acidity. This difference was entirely attributable to a difference between the two measures during the nocturnal period. Nocturnal gastric acid breakthrough was not a useful predictor of pathologic nocturnal esophageal reflux.

**Conclusion:**

In GERD subjects treated with a PPI, measuring time esophageal pH < 4 will significantly overestimate the prevalence of pathologic esophageal acid exposure over 24 hours and during the nocturnal period.

## Background

In 2004, a group from Stanford University reported that in asymptomatic GERD patients who were being treated with a PPI, 50% had pathologic esophageal acid exposure [1]. The authors of this paper stated that they were surprised by this high prevalence of pathologic esophageal acid exposure and an accompanying editorial pointed out that the findings were difficult to reconcile with a large body of evidence supporting the outstanding therapeutic efficacy of PPIs in GERD [2].

Previously [3], some of the present authors had reported that measuring time esophageal pH < 4 underestimates the effect of a PPI on esophageal acid exposure compared to measuring esophageal acid exposure as integrated esophageal acidity. We considered the possibility that the high prevalence of pathologic esophageal reflux in the Stanford study might simply have resulted from calculating acidity as time pH < 4. As a result, one of the present authors (JDG) contacted one of the authors from Stanford (GT) and proposed a collaboration that would involve calculating both integrated acidity and time pH < 4 from the original pH records. The present paper reports the results of these analyses as well as analyses of results from another study of GERD subjects that were conducted to test hypotheses that were generated from the Stanford data.

In analyses of both studies we found that calculating time esophageal pH < 4 substantially overestimates the prevalence of pathologic esophageal reflux compared to that determined by calculating integrated esophageal acidity. This difference, in turn, resulted from time pH < 4 overestimating the prevalence of pathologic nocturnal esophageal acidity.

We wondered if the overestimation of the prevalence of pathologic esophageal reflux might affect the apparent relationship between gastric acidity and pathologic esophageal reflux observed in the original Stanford report [1]. Accordingly, we examined relationships between gastric acidity and pathologic esophageal reflux in the original Stanford study and in another study of GERD subjects treated with a PPI using both time gastric pH < 4 and integrated gastric acidity.

## Methods

This report is based on data from 3 separate studies that have been described in detail previously [1,4].

The Stanford study (index study) involved 49 symptomatic GERD subjects who were treated with a PPI until they were asymptomatic. At the end of this period, gastric pH and esophageal pH were recorded continuously for 24 hours. The details of this study including a description of the pH recordings have been reported previously [1]. In the present analyses, 3 records were omitted because of technical inadequacies. Data from this study were used to compare the prevalence of pathologic esophageal reflux defined using time pH < 4 and to that defined using integrated acidity.

The 2nd study involved 26 healthy adults with no history of gastrointestinal disease or symptoms [4]. In these subjects, 24-hour gastric pH and esophageal pH were measured on 2 separate occasions, 7 days apart.

The 3rd study involved 57 adults with a history of GERD who experienced heartburn at least 4 times per week for at least 6 months [4]. In these subjects, 24-hour gastric pH and esophageal pH were measured once. Data from the second and third studies were used to establish cut-points that define pathologic esophageal reflux measured as time pH < 4 and as integrated acidity. These cut-points were then used to define pathologic esophageal reflux in the index study.

Also, in the 3rd study, 26 GERD subjects with esophageal pH < 4 for at least 10% of the 24-hour baseline recording period were randomized to receive 8 consecutive daily doses of 20 mg omeprazole or 20 mg rabeprazole in a crossover fashion with a 14-day washout between treatment periods. Gastric pH and esophageal pH were measured for 24 hours on days 1, 2 and 8 with each treatment. Analyses of data from Day 8 in the 3rd study were used to attempt to confirm results from the index study that compared pathologic esophageal reflux defined by time pH < 4 to that defined by integrated acidity.

For all pH recordings, subjects fasted from approximately 22:00 the evening before until the beginning of pH recording the following morning at 8:00. Gastric pH and esophageal pH values were recorded every 4 seconds using an ambulatory, dual channel pH recording system (Medtronic Synectics) with antimony electrodes (Zinetics24 single-use, internal-standard pH catheter). One electrode was placed in the esophagus 5 cm above the manometrically defined upper border of the lower esophageal sphincter. The other electrode was placed in the stomach 10 cm below the manometrically defined upper border of the lower esophageal sphincter (second and third studies) or 5 cm below the diaphragm determined by chest radiograph (index study). Electrodes were calibrated to pH 1 and 7, and connected to a portable data storage unit (Digitrapper, Medtronic Synectics). Recordings began at 8:00 and continued for 24 hours. Data were transferred from the portable data storage unit and processed using software designed for pH recordings (Polygram for Windows, Version 2.04, Medtronic Synectics). Raw data were exported from this software and used to calculate integrated acidity and time pH < 4.

Recently, Medtronic notified customers that new temperature correction factors should be used for the Slimline pH catheter as well as for the Zinetics24 single-use, internal-standard pH catheter [5]. Subsequently, two studies demonstrated the impact of these new correction factors on measures of acidity calculated from pH recordings [5,6]. We have not rescaled the data for the present analyses so that readers will be able to compare the present results to those from previous analyses of these same data [1,4,7].

Gastric and esophageal pH were recorded every 4^th ^second for 24 hours. Integrated esophageal acidity was calculated as described previously [3]. Integrated acidity is the time-weighted average of the hydrogen ion concentration expressed as mmol/L. It is also equal to the area under the hydrogen ion concentration-time curve. In contrast to time esophageal pH < 4, integrated esophageal acidity fully quantifies esophageal acid exposure. For example, two different one-hour periods of esophageal pH of 1 and 3 will give identical values for time esophageal pH < 4. In contrast, integrated esophageal acidity calculated from one hour of pH 1 will be 100-times higher than that calculated from one hour of pH 3.

The 24-hour recordings were divided into postprandial (9:00–22:00) and nocturnal (22:00–9:00) periods. As reported previously [4], the duration of the postprandial period was selected as 9:00–22:00, because integrated gastric acidity over this period gave the optimal correlation with meal-stimulated gastric acid secretion.

Bayes' rule was used to calculate the posterior probability that the prevalence of pathologic esophageal reflux represented false positive values [8].

Posterior Probability = Prior Probability × Likelihood/Total Probability

The likelihood value is the probability of the data for a given proportion and was calculated for values of proportions from 0 to 1.0 in steps of 0.05 using the following equation.

Likelihood = (proportion)^a ^× (1-proportion)^b^

where a is the number of subjects with pathologic esophageal reflux and b is the number of subjects without pathologic esophageal reflux.

The total probability is the weighted average of the various conditional probabilities calculated as

Total Probability = SUM [(Prior Probability)_i _× (Likelihood)_i_]

Where (Prior Probability)_I _is the ith prior probability and (Likelihood)_I _is the corresponding ith likelihood.

By dividing by total probability, all posterior probabilities are adjusted so that they sum to 1.0.

Statistical analyses were performed using GraphPad for InStat version 3.01 for Windows software.

## Results

Previously [7], we used data from the 2nd and 3rd studies to establish cut-points for 24-hour integrated esophageal acidity and time esophageal pH < 4 that had optimal sensitivity and specificity for distinguishing between normal and GERD subjects. In the present analyses we used these cut-points (8.1 mmol.hr/L for integrated acidity and 4.3% for time pH < 4) to compare the prevalence of pathologic esophageal reflux defined by the different measures of esophageal acidity in the index study.

**Figure 1:**
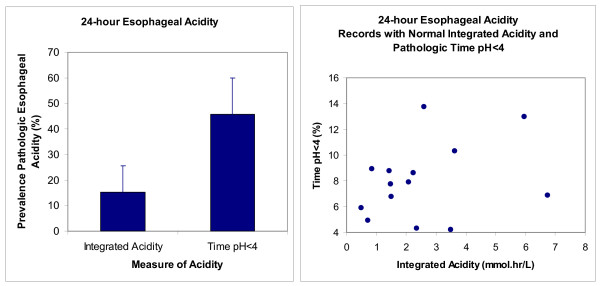
**Prevalence of pathologic 24-hour esophageal acidity assessed with integrated acidity and time pH < 4 in the index study (n = 46)**. The left panel gives prevalence with the 95% confidence interval. The right panel gives values for time pH < 4 from records with normal integrated acidity.

Figure 1-left illustrates that the prevalence of pathologic 24-hour esophageal reflux in the index study was significantly higher (P = 0.0005, McNemar's Test) when measured as time pH < 4 than when measured as integrated acidity. Figure 1-right shows that 14 normal values of integrated acidity had corresponding values of pathologic time pH < 4 as high as 14%.

We were interested in examining whether the differences in prevalence of pathologic 24-hour esophageal acidity occurred during the postprandial period, the nocturnal period or both. Before we could compare integrated esophageal acidity and time pH < 4 during the postprandial and nocturnal periods, it was necessary to determine cut-points that define pathologic esophageal reflux during the postprandial and nocturnal periods. We are unaware of any published values for integrated esophageal acidity that define pathologic postprandial or nocturnal esophageal reflux. Values for time esophageal pH < 4 that define pathologic postprandial and nocturnal esophageal acid exposure have been published [9-14]; however, we wanted to determine cut-points for the 2 different measures of acidity using the same cohorts of subjects and the same criterion. We determined these cut-points using receiver operating characteristic (ROC) analyses of data from the 2nd and 3rd studies, and selected the values for postprandial and nocturnal integrated esophageal acidity and time esophageal pH < 4 that had optimal sensitivity and specificity for distinguishing between normal and GERD subjects (Table 1).

**Table 1:** Cut-points, Sensitivities, Specificities and ROC Areas for Pathologic Integrated Esophageal Acidity and Time Esophageal pH < 4.

Period	Integrated Acidity (mmol.hr/L)	Time pH < 4(%)
**24-hour (900–900)**		
Cut-point	8.1	4.3
Sensitivity (%)	72	89
Specificity (%)	88	81
ROC Area	0.86 [0.77,0.94]	0.90 [0.84, 0.97]
**Postprandial (900–2200)**		
Cut-point	1.85	6.8
Sensitivity (%)	92	85
Specificity (%)	62	85
ROC Area	0.82 [0.72, 0.91]	0.88 [0.80, 0.95]
**Nocturnal (2200–900)**		
Cut-point	1.3	2.1
Sensitivity (%)	79	81
Specificity (%)	77	85
ROC Area	0.79 [0.69, .90]	0.84 [0.74, .92]

Results are from 26 normal and 57 GERD subjects. Values in brackets for ROC area are the 95 % confidence intervals.

Table 1 summarizes the cut-points for pathologic 24-hour, postprandial and nocturnal esophageal acidity assessed using integrated acidity and time pH < 4. For each analysis, the area under the ROC curve was significantly different from 0.5 (P < 0.0001). The cut-points were based on comparisons of data from all GERD subjects (n = 57) to data from all normal subjects (n = 26).

**Figure 2:**
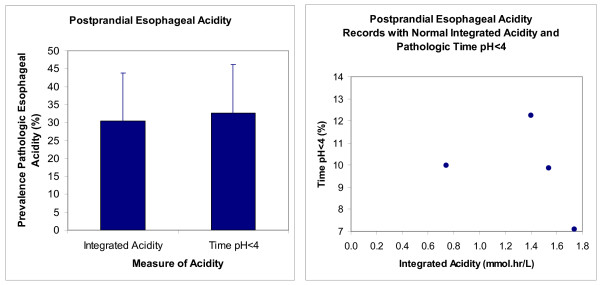
**Prevalence of pathologic postprandial esophageal acidity assessed with integrated acidity and time pH < 4 in the index study (n = 46)**. The left panel gives prevalence with the 95% confidence interval. The right panel gives values for time pH < 4 from records with normal integrated acidity.

Figure 2-left illustrates that the prevalence of pathologic postprandial esophageal reflux in the index study measured as time pH < 4 was not significantly different (P = 1.00, McNemar's Test) from that measured as integrated acidity. Figure 2-right shows that only 4 normal values of postprandial integrated acidity had corresponding values of pathologic time pH < 4.

**Figure 3:**
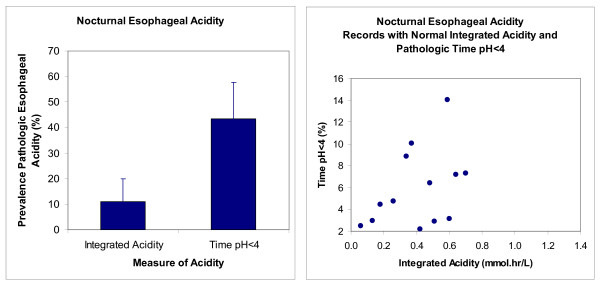
**Prevalence of pathologic nocturnal esophageal acidity assessed with integrated acidity and time pH < 4 in the index study (n = 46)**. The left panel gives prevalence with the 95% confidence interval. The right panel gives values for time pH < 4 from records with normal integrated acidity.

Figure 3-left illustrates that the prevalence of pathologic nocturnal esophageal reflux in the index study was significantly higher (P = 0.0003, McNemar's Test) when measured as time pH < 4 than when measured as integrated acidity. Figure 3-right shows that 13 normal values of integrated acidity had corresponding values of pathologic time pH < 4 as high as 14%.

**Figure 4:**
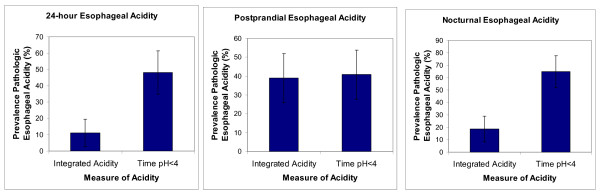
**Prevalence of pathologic esophageal acidity assessed with integrated acidity and time pH < 4 in the 3rd study (n = 52 records)**. Data are from 24-hour esophageal recordings on the 8^th ^day of treatment with 20 mg omeprazole or 20 mg rabeprazole. Vertical bars give the 95% confidence interval.

Figure 4 illustrates that results from the 3rd study confirm those from the index study. That is, the prevalence of both pathologic 24-hour and nocturnal esophageal reflux in the 3rd study was significantly higher (both P < 0.0001, McNemar's Test) when measured as time pH < 4 than when measured as integrated acidity. As was also the case with the index study, the prevalence of pathologic postprandial esophageal reflux in the 3rd study measured as time pH < 4 was not significantly different (P = 0.267, McNemar's Test) from that measured as integrated acidity.

The specificities for the cut-points given in Table 1 are all less than 100%; therefore, it is likely that at least some of the values for pathologic esophageal reflux during PPI treatment represent false-positive values. To examine this possibility, we used Bayes' rule to calculate the probability that the observed prevalence of pathologic esophageal reflux in the index and 3rd studies combined is greater than the prevalence of false-positive values. In Table 1, the specificity using the cut-point for pathologic 24-hour integrated esophageal acidity of 8.1 mmol.hr/L was 88% because 23 of the 26 normal subjects had values for 24-hour integrated esophageal acidity that were lower than 8,1. The 3 subjects with values above 8.1 (12%) represented false positives. In the index and 3rd study, 13% of subjects had pathologic integrated esophageal acidity and we used Bayes' rule to calculate the probability that this proportion was higher than the proportion of false positives in the control group used to establish the cut-point given in Table 1. Table 2 indicates that with 24-hour integrated esophageal acidity, the probability that the prevalence of pathologic esophageal reflux is greater than the corresponding prevalence of false-positive values is 0.139. Similarly, in Table 1, the specificity using the cut-point for pathologic time esophageal pH < 4 of 4.3% was 81% because 21 of the 26 normal subjects studied had values for 24-hour time esophageal pH < 4 that were lower than 4.3. The 5 subjects with values above 4.3 (19%) represented false positives. In the index and 3rd study, 47% of subjects had pathologic time esophageal pH < 4 and Table 2 indicates that the probability that the prevalence of 24-hour pathologic esophageal reflux is greater than the corresponding prevalence of false-positive values is 0.985. Corresponding probabilities for the nocturnal period were pathologic integrated acidity, 0.066, and time pH < 4, .999. Thus, the probability is at least 0.86 that values for pathologic integrated esophageal acidity are false-positive values, whereas the probability is at most only 0.015 that values for pathologic time esophageal pH < 4 are false-positive values.

**Table 2:** Bayesian Posterior Probabilities that the Prevalence of Pathologic Esophageal Reflux During PPI Treatment is Greater than the Prevalence of False-Positive Values for Pathologic Integrated Esophageal Acidity and Time Esophageal pH < 4.

	Probability that Observed Prevalence of Pathologic Esophageal Reflux is Greater than Prevalence of False-Positive Values	
**Period**	**Integrated Esophageal Acidity**	**Time Esophageal pH < 4**

24-hour	0.139	0.985
Nocturnal	0.066	0.999

Bayesian posterior probabilities were calculated using the values for the prevalence of false-positive values given in Table 1, the combined prevalence of pathologic esophageal reflux calculated from the index and 3rd studies, and a flat prior probability [8]. The calculations were for values of prevalence from 0 to 1.0 in steps of 0.05. The flat prior probability considered all prevalence values from 0 to 1.0 to be equally probable.

Table 3 gives the values for the area under the receiver operator characteristic (ROC) curves for analyses of gastric acidity stratified in terms of pathologic esophageal acid exposure. The area under the ROC curve is conceptually equivalent to the Wilcoxon statistic [15-18] and is a quantitative measure of the extent to which integrated gastric acidity differs between records with pathologic esophageal reflux and those with normal esophageal reflux. That is, the ROC area represents the probability that a subject selected randomly from the pathologic reflux group will have a higher value for gastric acidity than a subject selected randomly from the normal reflux group [15-18]. If there is no overlap in gastric acidity between the 2 groups, the area will be 1.00 and the probability will be 100%. If the distributions are identical, the area will be 0.50 and the probability will be 50%, i.e. the same as chance alone.

**Table 3:** Parameter Values for the Relationship Between Measures of Gastric Acidity and the Odds Pathologic Esophageal Reflux.

	**Index Study (n = 46 records)**		**3rd Study (n = 156 records)**	
Period	Integrated Acid (mmol.hr/L)	Time pH < 4 (%)	Integrated Acid (mmol.hr/L)	Time pH < 4 (%)

**24-hour (900–900)**				
ROC Area	0.77 (0.59, 0.94)	0.68 (0.52, 0.83)	0.74 (0.66, 0.82)	0.64 (0.55, 0.74)
Overall Odds Pathologic Reflux	0.18	0.77	0.37	2.52
**Postprandial (900–2200)**				
ROC Area	0.71 (0.55, 0.88)	0.69 (0.52, 0.85)	0.80 (0.74, 0.87)	0.77 (0.70, 0.85)
Overall Odds Pathologic Reflux	0.44	0.48	1.05	1.31
**Nocturnal (2200–900)**				
ROC Area	0.75 (0.57, 0.93)	0.62 (0.45, 0.78)	0.75 (0.67, 0.83)	0.62 (0.53, 0.71)
Overall Odds Pathologic Reflux	0.18	0.84	0.21	1.59

Parameter values were calculated from data illustrated in Figures 1–4 using values for gastric acidity stratified on the basis of pathologic esophageal acidity. Numbers in parentheses for ROC area give the 95% confidence interval.

In the index study, the ROC areas for integrated acidity were all higher than 0.7, and each curve was significantly different from 0.5. In contrast, for the data for time pH < 4, the ROC areas were all lower than 0.7, and the area for nocturnal gastric acidity in the index study was not significantly different from 0.5 (Table 3). The overall odds of pathologic time esophageal pH < 4 were higher than corresponding values for pathologic integrated esophageal acidity over 24 hours and during the nocturnal period (Table 3), because in GERD subjects treated with a PPI, measuring time esophageal pH < 4 significantly overestimates the prevalence of pathologic esophageal acid exposure during these periods.

To attempt to confirm the results from the index study, we conducted similar analyses of data from days 1, 2 and 8 of PPI treatment in the 3rd study. We anticipated that we might obtain more precise estimates of relationships between gastric acidity and the probability of pathologic esophageal reflux with data from the 3rd study, because more records (156) would be analyzed and the data would be distributed over a wider range because on days 1 and 2, treatment effects of the PPI would be submaximal. This latter phenomenon was supported by the finding that the overall odds of pathologic esophageal reflux for each period in the 3rd study were higher than corresponding values from the index study (Table 3).

A major feature of analyses of results from the 3rd study is that they confirmed corresponding results from the index study. That is, these analyses showed that both integrated gastric acidity and time gastric pH < 4 provide important information regarding the probability of pathologic esophageal reflux during the same period. One difference, however, is that in the 3rd study, the ROC curve for time gastric pH < 4 for the nocturnal period was significantly different from chance alone, because the 95% confidence interval for the ROC area does not include 0.5 (Table 3). This finding indicates that the lack of statistical significance for corresponding analyses from the index study probably represents a false-negative value because of the smaller sample size in the index study.

As occurred in the index study, in the 3rd study, the ROC areas with time pH < 4 were consistently lower than corresponding values with integrated acidity, and the overall odds of pathologic esophageal reflux with time pH < 4 were consistently higher than corresponding values with integrated acidity (Table 3).

Some have evaluated gastric pH during PPI treatment by measuring nocturnal gastric acid breakthrough (NAB; 19–24). Initially, NAB was arbitrarily defined as continuous nocturnal gastric pH <4 for more than 1 hour in subjects who received a PPI twice daily [19-21]. Subsequently, however, NAB was defined as simply continuous nocturnal gastric pH <4 for more than 1 hour during PPI treatment [25]. Although NAB has been claimed to increase the risk of nocturnal esophageal acid reflux [25], none of the published analyses have shown clearly that NAB distinguishes between normal and pathologic nocturnal esophageal reflux during the same time and in the same subjects in which NAB occurs. Accordingly, we examined the extent to which NAB might be useful in predicting the occurrence of pathologic nocturnal esophageal reflux in GERD subjects during PPI treatment.

Table 4 indicates that in the index study, 34 of 46 subjects (74%) had NAB. Of the subjects with NAB, only 7 (21%) had pathologic nocturnal integrated esophageal acidity and 18 (53%) had pathologic nocturnal time esophageal pH < 4. In the 3rd study, 140 of 156 subjects (90%) had NAB. Of the subjects with NAB, only 49 (35%) had pathologic nocturnal integrated esophageal acidity and 97 (69%) had pathologic nocturnal time esophageal pH < 4. As mentioned previously, the higher percentages of pathologic esophageal acidity with time pH < 4 compared to those with integrated acidity occur because time pH < 4 overestimates the prevalence of pathologic esophageal acidity.

**Table 4:** Prevalence of Pathologic Esophageal Reflux Stratified by Nocturnal Gastric Breakthrough.

	**Index Study**			
	NAB (n = 34)		No NAB (n = 12)	

Pathologic Nocturnal Esophageal Acid	Integrated Acidity	Time pH < 4	Integrated Acidity	Time pH < 4

YES	7	18	0	3
NO	27	16	12	9

	**3^rd ^Study**			

	NAB (n = 140)		No NAB (n = 16)	

Pathologic Nocturnal Esophageal Acid	Integrated Acidity	Time pH < 4	Integrated Acidity	Time pH < 4

YES	49	97	1	9
NO	91	43	15	7

Table 4 also indicates that if NAB is absent there is a high probability that integrated nocturnal esophageal acidity will be normal, because of the subjects without NAB 0 of 12 subjects in the index study and 1 of 16 subjects in the 3rd study had pathologic integrated esophageal acidity. This is not the case, however, measuring time esophageal pH < 4.

## Discussion

Previously [3], we found that the effect of rabeprazole on esophageal acid exposure was significantly less when measured as time pH < 4 than when measured as integrated acidity. The present analyses illustrate an important clinical consequence of this difference; namely, in asymptomatic GERD subjects being treated with a PPI, 46% had pathologic 24-hour esophageal acid exposure measured as time pH < 4, whereas only 15% of the same records had pathologic esophageal acid exposure measured as integrated acidity. The possibility that the surprisingly high prevalence of pathologic esophageal reflux in asymptomatic GERD subjects during PPI treatment was an artifact resulting from the way that the data were analyzed was not considered by the authors of the original paper [1] or the accompanying editorial [2].

It is important to emphasize that the major difference between integrated acidity and time pH < 4 relates to the prevalence of pathologic esophageal reflux. If esophageal reflux is pathologic with time pH < 4, there is a low probability that it will also be pathologic with integrated acidity. On the other hand, if esophageal acid exposure is normal with time pH < 4, there is a high probability that it will also be normal with integrated acidity.

Our definition of pathologic esophageal reflux is based on the value for esophageal acidity (integrated acidity or time pH < 4) that best distinguishes between normal and GERD subjects. Others have measured pathologic esophageal reflux as those reflux episodes that are associated with symptoms attributable to GERD [26]. This definition of pathologic esophageal reflux may be helpful in understanding the pathogenesis of symptoms such as heartburn, but it is not useful in distinguishing between normal and GERD subjects on the basis of esophageal acid exposure.

The significant difference in the prevalence of pathologic 24-hour esophageal reflux measured as integrated acidity compared to that measured as time pH < 4 was attributable to a difference between the two measures during the nocturnal period. The prevalence of pathologic esophageal reflux during the postprandial period was similar with integrated acidity and time pH < 4. In contrast, the prevalence of pathologic esophageal reflux during the nocturnal period was significantly lower with integrated acidity than with time pH < 4. This difference during the nocturnal period is probably attributable to long periods of esophageal pH between pH 3 and pH 4, which will give relatively high values for time pH < 4, but relatively low values of integrated acidity.

Another important difference between the two measures of esophageal acidity is that with integrated acidity, the PPI is much more effective during the nocturnal period than during the postprandial period. In contrast, with time pH < 4, the prevalence pathologic esophageal reflux is similar during the nocturnal and postprandial periods. This difference with integrated acidity is consistent with the findings in GERD subjects with esophagitis who are treated with a PPI that esophagitis heals in approximately 90%, while heartburn resolves in only 60–70% [27]. It seems likely that abolishing pathologic nocturnal esophageal acid is important in healing esophagitis, whereas abolishing pathologic postprandial esophageal acid is important in resolving heartburn. Measuring time esophageal pH < 4 cannot account for these findings in GERD subjects with esophagitis.

All of the major findings from analyses of the index study were replicated in analyses of the 3rd study. This confirmation indicates that the findings in the index study reflect reproducible differences between integrated esophageal acidity and time esophageal pH < 4 in GERD subjects treated with a PPI, and do not result from unrecognized peculiarities related to the conduct of the index study.

The results from the present analyses of esophageal pH from GERD subjects may also be applicable to subjects with Barrett's esophagus, who are believed to be relatively resistant to PPI treatment [28-30]. A substantial portion of subjects with Barrett's esophagus have been found to have pathologic esophageal acid exposure even when treated with high doses of a PPI. In all instances, however, esophageal acid exposure has been measured as time esophageal pH < 4 [17-19], and as shown in the present analyses, this measurement leads to artifactually high estimates of the prevalence of pathologic esophageal reflux.

From the clinical standpoint, our present findings only affect the interpretation of measurements of 24-hour esophageal pH; they do not influence the indications for, such measurements. For example, if a clinician records esophageal pH in a patient with GERD to assess the effect of a PPI on esophageal acid exposure, integrated esophageal acidity should be calculated instead of time esophageal pH < 4, because this will reduce the possibility of reaching a false conclusion that esophageal acid exposure is abnormally high.

Our present analyses also indicate that NAB [19-25] is not a useful indicator of the probability of pathologic nocturnal esophageal reflux. Using integrated esophageal acidity, most subjects with NAB did not have pathologic esophageal reflux. On the other hand, although the absence of NAB was infrequent in GERD subjects during treatment with a PPI, when NAB was absent, it was a useful predictor of normal nocturnal integrated esophageal acidity. Using time esophageal pH < 4, both the presence and absence of NAB were associated with higher probabilities of pathologic esophageal reflux.

Our conclusion that NAB is not a useful predictor of pathologic esophageal reflux agrees with the conclusion from a previous study that measured gastric and esophageal pH in normal and GERD subjects at baseline and during PPI treatment [24]. Although this previous study did not relate NAB to nocturnal esophageal pH measured at the same time, the data provided appear to indicate that for NAB in GERD subjects during PPI treatment, its positive predictive value was low and its negative predictive value high.

One limitation that applies to analyses of gastric pH recordings in general, is that they do not consider the effect of the PPI on gastric volume. This is potentially important because the effect of a PPI during fasting is primarily on gastric volume with only a small change in gastric pH [31,32]. This phenomenon would result in prevention of pathologic nocturnal esophageal reflux by a relatively small decrease in gastric acidity accompanied by a decrease in intragastric volume. Even with this caveat, however, measuring gastric acidity during the nocturnal period still provides important information regarding the likelihood of pathologic nocturnal esophageal reflux.

## Conclusion

In GERD subjects treated with a PPI, measuring time esophageal pH < 4 will significantly overestimate the prevalence of pathologic esophageal acid exposure over 24 hours and during the nocturnal period.

## Competing interests

Jerry Gardner is President of Science for Organizations. Winston Young is President of Blossomtech. Sheldon Sloan was an employee of Janssen Pharmaceutical Research Foundation.

## Authors' contributions

All authors reviewed, edited and approved the final version of the manuscript. LBG, GT and JDG designed the analyses. GT and PS conducted the index study and provided the raw data from the pH recordings. SS, MR and PBM designed and conducted the 2^nd ^and 3^rd ^studies, and PBM provided the raw data from the pH recordings. WY processed the raw data from the pH recordings and provided statistical advice. JDG preformed the analyses and wrote the paper.

## Pre-publication history

The pre-publication history for this paper can be accessed here:


